# Association of medication administration errors with interruption among nurses in public sector tertiary care hospitals

**DOI:** 10.12669/pjms.35.5.287

**Published:** 2019

**Authors:** Raja, Badil, Sajid Ali, Shaheen Sherali

**Affiliations:** 1 Raja, MS. Nursing. Staff Nurse, Department of Plastic and Reconstructive Surgery, Dr. Ruth K.M. Pfau, Civil Hospital, Karachi, Pakistan; 2 Badil, MS. Nursing. Assistant Professor, Institute of Nursing, Dow University of Health Sciences, Karachi, Pakistan; 3 Sajid Ali, BSc. Nursing. Lecturer, Liaquat National College of Nursing, Karachi, Pakistan; 4 Shaheen Sherali, MS. Nursing. Vice Principal, Indus College of Nursing & Midwifery, Karachi, Pakistan

**Keywords:** Medication Administration Errors, Interruption, Nurses, Tertiary Care Hospitals

## Abstract

**Objectives:**

To determine the association of medication administration errors with interruption among nurses working at public sector tertiary care hospitals in Karachi, Pakistan.

**Methods:**

An analytical cross-sectional study was accomplished at two public sector healthcare facilities Civil Hospital, and Dow University Hospital, Karachi. The study was carried out from October 2017 to July 2018 over a period of 10 months. The sample was calculated by using OpenEpi version 3.0. By taking 56.4% of medication administration errors, 5% margin of error and 95% confidence level. The calculated sample size was 204 of both genders. The subjects both male and female nurses having a valid license from Pakistan Nursing Council and one year of clinical experience were enrolled in the study. The subjects were approached by using non-probability purposive sampling method. Validated and adapted questionnaire utilized to gather the data. Data was entered and analyzed by using SPSS version 21.0.

**Results:**

In this study, total 204 nurses were included, almost half (52%) of them were male. Majority of (82.3%) study participants had age between 25-35 years old. There were total 716 medications given by 204 nurses. Out of these, 295 (41.2%) were antibiotics, other common medications were acid-suppressive, analgesic and antiemetic 14.5%, 15.9% and 11.2% respectively. Among all 716 medications, 644 (89.9%) were given intravenously whereas only 6.7% drugs given orally. A significant association has been found between medication administration errors and interruption like talking with other health care personnel, patients or attendant queries, phone calls (p-value=<0.001). Nearly 91% of the study nurses who were interrupted during medication committed medication errors.

**Conclusion:**

It is concluded that there is a significant association between medication administration errors with interruption among nurses.

## INTRODUCTION

In every health care sector, a large number of medications are administered to the patients every day. In this whole process medication errors are most common.^1^ The process of medication in the hospital setup comprises of five phases including medication prescription, preparation, dispensation, administration and monitorization. Each phase of this process may cause risk for medication errors.^2^ Furthermore, it is documented by current research that interruptions and distractions are the major risk factors which are contributing to medication administration errors.^3^ Moreover, current research studies have confirmed that interruptions at work place are very common. On other hand, it is usually recognized as the norm.^4^ On account of administration of medication, a current research study showed that nurse’s encountered 57 interruptions out of 100.^5^ According to a study nurses who are working in hospital perceive that there are many causes of medication administration errors like heavy workload, insufficient knowledge, lack of following policy and procedures, improper communication, different system issues, differences of standards of training, anxiety, deviation of attention, interruptions, distractions, plus poor staffing are similarly cited.^6^-^8^

Before a decade medication administration and interruption received very limited research attention, despite of having clear evidences demonstrating serious harm to the patient.^9^ Since last 10 years there is steady progress research work on reporting intervention when nurses prepare the medication for administration to patients. Furthermore, it is revealed by latest research study that almost 7,000 doses are administered per day in England. If these doses administered 99.9% accurately even than there are chances of seven errors per day, so nurses have to gain theoretical and numerical knowledge pertaining to pharmacology.^10^ It is evident by research that about 20% of all medication administration results in error. Among all reported errors, only two errors per day remain unreported.^11^

It is revealed that nurses have short time to focus on medication administration; there are more chances of frequent interruption. It is also found that the interruption is caused by other health care personals for seeking assistance; phone calls, system failure, and access towards equipment were found to be those interruptions which are avoidable.^12^ Although, medication administration errors lead to prolong hospitalization, over cost and serious harm to the patient’s.^13^ Thus, this research was conducted in order to determine the association of medication administration errors with interruption among nurses working at public sector tertiary care hospitals, Karachi.

## METHODS

An analytical cross-sectional study was accomplished at two public sector organizations, Civil Hospital and Dow University Hospital, Karachi. The study was carried out from October 2017 to July 2018 over a period of 10 months. The sample was calculated by using OpenEpi version 3.0. By taking 56.4% of medication administration errors, 5% margin of error and 95% confidence level. The calculated sample size was 204 of both genders. The subjects both male and female nurses having valid license from Pakistan Nursing Council and one year of clinical experience were enrolled in the study. The subjects were approached by using non-probability purposive sampling method. Validated and adapted questionnaire utilized to gather the data.

Data was collected after approval from Institutional Review Board (IRB) of Dow University of Health Sciences (DUHS). Furthermore, permission was also granted from Medical Superintendent of CHK and DUH Karachi. Subject’s participation was voluntary. Confidentiality of the data was also assured.

### Data analysis

Data was entered and analyzed by using SPSS version 21.0. Data was shown in mean ± standard deviation for all quantitative variables. Data was also analyzed in frequency and percentages for all qualitative variables like designation, gender, educational status, working area of nurses, hospital, interruption from the time of medication preparation to administration, and duty shift.Chi-squire test was used to identify the significance association of interruption with medication administration error. Level of significance was considered as ≤0.05.

## RESULTS

In this study 204 nurses were included, almost half (52%) of them were male. Majority (82.3%) of the study participants had age between 25-35 years old. In addition, 65.2% of the participants had diploma in general nursing. Approximately half (47.5%) study participants had experience below five years. More than half (53.4%) participants were working in morning shift. While, 69.1% of the study participants faced interruption while administrating medication to the patients. A total of 204 patients with mean age of 35.71 were included while the nurse administered their medication.

The distribution of type of the drugs given by nurses is shown in Table-I. There were a total of 716 drugs given by 204 nurses. Out of these drugs, 295 (41.2%) were antibiotics, other prominent drugs were acid-suppressive, analgesic and antiemetic 14.5%, 15.9% and 11.2% respectively.

**Table I:** Distribution of observed drugs (n = 716).

Type of the drugs	N	%
Antibiotic	295	41.2%
Acid-suppressive drugs	104	14.5%
Analgesic	114	15.9%
Antiemetic	80	11.2%
Antihistamine	1	0.1%
Micro & Macro nutrients Appetizer	51	7.1%
Anti-Diabetic	2	0.3%
Anticoagulant - Antiplatelet drugs	10	1.4%
Antihypertensive drugs	9	1.3%
Epileptic	2	0.3%
Anti-inflammatory drug	21	2.9%
Tranquilizer	2	0.3%
Anti-malarial	2	0.3%
Anti-lipid	1	0.1%
Others	22	3.1%

The distribution of route of administration is shown in Table-II. Among all 716 drugs given by the nurses, 644 (89.9%) were intravenous whereas only 6.7% drugs given orally.

**Table II:** Distribution of route of observed medication (n = 716).

Route of administration	N	%
Intravenous	644	89.9%
Intramuscular	12	1.7%
Subcutaneous	5	0.7%
Inhalation	6	0.8%
Oral	48	6.7%
Local application	1	0.1%

The association of medication administration errors like missed dose errors, wrong route errors, wrong time errors, wrong dose errors, improper administration errors, and inappropriate documentation with interruption such as information exchanges between nursing staff, management of telephone calls and patients or attendant queries, noise is shown in Table-III. This table shows that more interrupted nurses committed medication administration errors as compared to those who were not interrupted, this showed a significant association with medication administration errors and interruption (p-value=<0.001). Nearly 91% of the nurses who were interrupted committed medication errors.

**Table III:** Association of medication administration error with interruption.

	No Medication administration error		Medication administration error		Chi	P-value

	N	%	N	%		
Interruption					24.45	<0.001
Yes	13	9.2%	128	90.8%		
No	24	38.1%	39	61.9%		

## DISCUSSION

Medication administration is one of the key responsibilities among all other responsibilities which nurses perform while they are taking care of their patients. Therefore, it requires high attention while performing medications administration. It is reported that among all kinds of clinical error, MAE is a 2^nd^ most common cause of injury.^14^ Literature has proven that interruptions during medications administration lead to medications errors which can lead to fatal or other complications.^15^

In our study, the results show that 90.8% of the participants faced some kind of interruption like queries handling, providing information to student nurses, supervisors round, and seeking assistants by other healthcare personnel’s, recently prescribed medications, shortage of medication patient demands while medicating their patients. This result is higher than other study conducted in Canada (59.1%)^16^ and Australia (53.1%).^17^

We found 53.4% participants were working in morning shift in week days faced interruption, this result contradicts with other study showing nurses who work in weekend face major interruptions.^18^ This is because our nurses aren’t only given the task for medications but, also have to look over their other tasks e.g. prepare patients for any procedure, answer phone calls, respond to patients’ visitors and other patients, attend doctor rounds – These are all the activities that nurses are performing during the morning shift.

This is proven in our study that nurses who encounter more interruption during medication are committing way more medication errors as compared to their counterparts who weren’t. The error could be medication application, wrong route of medication, improper dosage, wrong medication given to wrong patient (which can be fatal)^19^-^21^, delay in treatment, can develop complication it can also effect wrong impression of nursing profession, mistrust, job insecurity, anxiety, stress etc. Now, it is time to take action, to prevent such types of errors. Our study suggests that by distributing staff workloads equally can help prevent this.^22^ One nurse should be separately assigned for medication administration, who will not be involved in any other care-taking. Staffs should be reinforced to double checking medication orders before administering the medication.

In this study 204 nurses administrated 716 medications, the majority 295 (41.2%) were antibiotics. Study conducted in Ethiopia reported that among 360 observed medications there were 77.8 % of the medications were antibiotics.^23^

The study conducted in Italy found following interventions effective to control interruption during medication administration, a visual notices, a yellow taped floor indicating the ‘No interruption area’, and a yellow ribbon worn by nurses during medication cycles. We can also replicate these interventions in our area also to decreased medications error.^2^

## CONCLUSION

There is statistically significant association between medication administration errors with interruption among nurses.

### Authors’ Contribution

**R:** Conceived idea, manuscript writing.

**B:** Statistical analysis, proof reading.

**SA:** Literature searching, contributed in manuscript writing.

**SS:** Data collection, critical review.
